# Association of Race With Mortality Among Patients Hospitalized With Coronavirus Disease 2019 (COVID-19) at 92 US Hospitals

**DOI:** 10.1001/jamanetworkopen.2020.18039

**Published:** 2020-08-18

**Authors:** Baligh R. Yehia, Angela Winegar, Richard Fogel, Mohamad Fakih, Allison Ottenbacher, Christine Jesser, Angelo Bufalino, Ren-Huai Huang, Joseph Cacchione

**Affiliations:** 1 Ascension Health, St Louis, Missouri; 2 Ascension Data Science Institute, St Louis, Missouri; 3 Ascension Clinical Research Institute, St Louis, Missouri

## Abstract

**Importance:**

While current reports suggest that a disproportionate share of US coronavirus disease 2019 (COVID-19) cases and deaths are among Black residents, little information is available regarding how race is associated with in-hospital mortality.

**Objective:**

To evaluate the association of race, adjusting for sociodemographic and clinical factors, on all-cause, in-hospital mortality for patients with COVID-19.

**Design, Setting, and Participants:**

This cohort study included 11 210 adult patients (age ≥18 years) hospitalized with confirmed severe acute respiratory coronavirus 2 (SARS-CoV-2) between February 19, 2020, and May 31, 2020, in 92 hospitals in 12 states: Alabama (6 hospitals), Maryland (1 hospital), Florida (5 hospitals), Illinois (8 hospitals), Indiana (14 hospitals), Kansas (4 hospitals), Michigan (13 hospitals), New York (2 hospitals), Oklahoma (6 hospitals), Tennessee (4 hospitals), Texas (11 hospitals), and Wisconsin (18 hospitals).

**Exposures:**

Confirmed SARS-CoV-2 infection by positive result on polymerase chain reaction testing of a nasopharyngeal sample.

**Main Outcomes and Measures:**

Death during hospitalization was examined overall and by race. Race was self-reported and categorized as Black, White, and other or missing. Cox proportional hazards regression with mixed effects was used to evaluate associations between all-cause in-hospital mortality and patient characteristics while accounting for the random effects of hospital on the outcome.

**Results:**

Of 11 210 patients with confirmed COVID-19 presenting to hospitals, 4180 (37.3%) were Black patients and 5583 (49.8%) were men. The median (interquartile range) age was 61 (46 to 74) years. Compared with White patients, Black patients were younger (median [interquartile range] age, 66 [50 to 80] years vs 61 [46 to 72] years), were more likely to be women (2259 [49.0%] vs 2293 [54.9%]), were more likely to have Medicaid insurance (611 [13.3%] vs 1031 [24.7%]), and had higher median (interquartile range) scores on the Neighborhood Deprivation Index (−0.11 [−0.70 to 0.56] vs 0.82 [0.08 to 1.76]) and the Elixhauser Comorbidity Index (21 [0 to 44] vs 22 [0 to 46]). All-cause in-hospital mortality among hospitalized White and Black patients was 23.1% (724 of 3218) and 19.2% (540 of 2812), respectively. After adjustment for age, sex, insurance, comorbidities, neighborhood deprivation, and site of care, there was no statistically significant difference in risk of mortality between Black and White patients (hazard ratio, 0.93; 95% CI, 0.80 to 1.09).

**Conclusions and Relevance:**

Although current reports suggest that Black patients represent a disproportionate share of COVID-19 infections and death in the United States, in this study, mortality for those able to access hospital care did not differ between Black and White patients after adjusting for sociodemographic factors and comorbidities.

## Key Points

**Question:**

Is race associated with mortality among patients hospitalized with coronavirus disease 2019 (COVID-19) in the United States?

**Findings:**

In this cohort study of 11 210 individuals with COVID-19 presenting for care at 92 hospitals across 12 states, there was no difference in all-cause, in-hospital mortality between White and Black patients after adjusting for age, sex, insurance status, comorbidity, neighborhood deprivation, and site of care.

**Meaning:**

In this study, race was not independently associated with in-hospital mortality after adjusting for differences in sociodemographic and clinical factors.

## Teaser

This cohort study evaluates the association of race with all-cause, in-hospital mortality among patients with coronavirus disease 2019, adjusting for sociodemographic and clinical factors.

## Introduction

As coronavirus disease 2019 (COVID-19) continues to spread across the United States, understanding how race is associated with outcomes will be important to clinicians, health systems, and public officials responding to this pandemic.^1,2^ Current reports suggest that a disproportionate share of US COVID-19 cases are among Black residents (21%) compared with their proportion of the US population (13%).^3^ However, much of our national-level data relies on aggregated local, state, and territorial reports to the US Centers for Disease Control and Prevention (CDC), which have high proportions (55%) of missing race/ethnicity data.^4^

In 20 of 31 states reporting COVID-19 cases by race/ethnicity, Black patients accounted for a greater share of confirmed cases compared with their share of the total population.^4,5^ Additionally, Black patients represented more than half of all COVID-19 deaths in Alabama (52%), Georgia (51%), Louisiana (59%), Mississippi (66%), and the District of Columbia (75%).^4,5^

To date, US studies evaluating the association of race with COVID-19 outcomes have mostly been limited to summary statistics, with large amounts of missing data or localization to specific states.^3,4,5,6,7,8,9,10,11,12^ We performed analyses on a large cohort of patients with COVID-19 to better understand the association between race and mortality, alone and in combination with age, sex, insurance status, comorbidity, and social factors, among patients who accessed hospital care for their disease.

## Methods

Adults (age ≥18 years) with confirmed severe acute respiratory syndrome coronavirus 2 (SARS-CoV-2) infection (positive result by polymerase chain reaction testing of a nasopharyngeal sample) presenting to Ascension hospitals between February 19, 2020, and May 31, 2020, were included. To fully capture hospital outcomes, patients admitted during this time period were observed through June 25, 2020. Hospitals were located in 12 states: Alabama (6 hospitals), Maryland (1 hospital), Florida (5 hospitals), Illinois (8 hospitals), Indiana (14 hospitals), Kansas (4 hospitals), Michigan (13 hospitals), New York (2 hospitals), Oklahoma (6 hospitals), Tennessee (4 hospitals), Texas (11 hospitals), and Wisconsin (18 hospitals). As a system, standard protocols were established and implemented for COVID-19 screening, testing, and management across the sites of care. The Ascension Seton institutional review board approved the study protocol as exempt and granted a waiver of consent because this retrospective study was based on secondary use of data arising from routine care. This study followed the Strengthening the Reporting of Observational Studies in Epidemiology (STROBE) reporting guideline.

Patient sociodemographic characteristics, laboratory results, and health care utilization, sourced from electronic health records and administrative data, were abstracted, quality assured, and assembled into a uniform database. Age was divided into the 4 following groups: 18 to 49 years, 50 to 64 years, 65 to 84 years, and 85 years and older. Race was self-reported and categorized as White, Black, and other and available for 98% of the patients included in the study. Insurance status was categorized as commercial, Medicare, Medicaid, other (eg, TRICARE, self-pay), and unknown. The Agency for Healthcare Research and Quality Elixhauser Comorbidity Index (ECI), a method of assessing comorbidities based on 29 groups of *International Statistical Classification of Diseases and Related Health Problems, Tenth Revision *(*ICD*-*10*) diagnosis codes, was calculated.^13,14^ The index ranged from −45 to 159 in our sample, with higher numbers indicating greater comorbidity. In addition, *ICD*-*10* diagnosis codes were used to assess the following coexisting conditions: asthma, cancer, chronic kidney disease, chronic liver disease, chronic obstructive pulmonary disease, congestive heart failure, coronary artery disease, diabetes, hypertension, obesity, and solid organ transplantation. Diagnosis codes used to calculate the ECI and examine the presence of coexisting conditions were drawn from hospital billing data for the patient encounters included in this analysis. If available, first documented temperature, respiratory rate, and oxygen saturation level were extracted from the electronic health record. Using each patient’s home address, we assigned the neighborhood deprivation index (NDI) for their zip code. The NDI is a composite of social and material deprivation composed of 8 variables collected in the American Community Survey focused on poverty, employment, education, and housing.^15^ The NDI ranged from −1.80 to 3.23 in our sample, with higher numbers indicating greater deprivation. We described summary statistics for patient sociodemographic characteristics, health care utilization (intensive care unit [ICU] stay, use of mechanical ventilation), and all-cause in-hospital mortality (indicated by use of hospital discharge status code 20, expired).

### Statistical Analysis

Cox proportional hazards regression with mixed effects was used to evaluate associations between patient characteristics and time to all-cause, in-hospital mortality by calculating hazard ratios (HRs) with 95% CIs. These models account for cluster-specific random effects that may result in differing baseline hazard functions between hospitals. We used 3 models to assess the association of race with mortality; all models included the random effects term for hospital. Model 1 estimated HRs for patient characteristics, unadjusted for other factors. Model 2 included a priori patient characteristics of interest (race, age, sex, insurance, and ECI and NDI scores). Model 3 adjusted for the covariates in model 2 and specific chronic conditions if they were significant in univariable analyses at *P* < .05. The proportional hazards assumption for the Cox models was assessed and confirmed graphically. Sensitivity analyses were performed to investigate potential residual confounding by modeling age as a categorical variable rather than as a continuous variable. Two-sided testing was used, with *P* < .05 considered statistically significant. Statistical analyses were performed using R version 3.6.2 (R Project for Statistical Computing).

## Results

Of 11 210 patients with confirmed COVID-19, 5583 (49.8%) were men. The median (interquartile range [IQR]) age was 61 (46 to 74) years, 4180 patients (37.3%) were Black individuals, 2591 patients (23.1%) were covered by Medicaid, and the median (IQR) ECI score was 17 (0-41) (Table 1). Compared with White patients, Black patients were younger (median [IQR] age, 66 [50 to 80] years vs 61 [46 to 72] years), more likely to be women (2259 [49.0%] vs 2293 [54.9%]), more likely to be covered by Medicaid (611 [13.3%] vs 1031 [24.7%]), and had greater median (IQR) NDI scores (−0.11 [−0.70 to 0.56] vs 0.82 [0.08 to 1.76]) and ECI scores (21 [0 to 44] vs 22 [0 to 46]). Compared with White patients, a greater proportion of Black patients had asthma (216 [4.7%] vs 367 [8.8%]), cancer (145 [3.2%] vs 151 [3.6%]), chronic kidney disease (595 [12.9%] vs 858 [20.5%]), congestive heart failure (496 [10.8%] vs 521 [12.5%]), diabetes (1061 [23.0%] vs 1337 [32.0%]), hypertension (1153 [25.0%] vs 1265 [30.3%]), obesity (838 [18.2%] vs 1345 [32.2%]), and solid organ transplantation (14 [0.3%] vs 21 [0.5%]).

**Table 1.** Characteristics of Adults With Coronavirus Disease 2019 Presenting to 92 Hospitals in a Multistate US Health Care System^a^

Characteristic	Patients, No. (%)			
	All (N = 11 210)	White (n = 4606)	Black (n = 4180)	With other or missing race (n = 2424)
Age, median (IQR), y	61 (46 to 74)	66 (50 to 80)	61 (46 to 72)	53 (41 to 67)
Sex				
Women	5622 (50.2)	2259 (49.0)	2293 (54.9)	979 (43.5)
Men	5583 (49.8)	2346 (50.9)	1886 (45.1)	1267 (56.3)
Missing	5 (0.04)	1 (0.02)	1 (0.02)	3 (0.13)
Insurance				
Commercial	2872 (25.6)	1191 (25.9)	1054 (25.2)	565 (25.1)
Medicare	4922 (43.9)	2449 (53.2)	1911 (45.7)	518 (23.0)
Medicaid	2591 (23.1)	611 (13.3)	1031 (24.7)	913 (40.6)
Other	531 (4.74)	193 (4.19)	114 (2.73)	204 (9.07)
Unknown	294 (2.62)	162 (3.52)	70 (1.67)	49 (2.18)
Comorbidities				
ECI, median (IQR)^b^	17 (0 to 41)	21 (0 to 44)	22 (0 to 46)	0 (0 to 31)
Asthma	628 (5.60)	216 (4.69)	367 (8.78)	41 (1.82)
Cancer	312 (2.78)	145 (3.15)	151 (3.61)	15 (0.67)
Chronic kidney disease	1542 (13.8)	595 (12.9)	858 (20.5)	77 (3.42)
Chronic liver disease	311 (2.77)	152 (3.30)	132 (3.16)	23 (1.02)
COPD	950 (8.47)	506 (11.0)	409 (9.78)	32 (1.42)
Congestive heart failure	1075 (9.59)	496 (10.8)	521 (12.5)	47 (2.09)
Coronary artery disease	1168 (10.4)	601 (13.0)	507 (12.1)	53 (2.36)
Diabetes	2585 (23.1)	1061 (23.0)	1337 (32.0)	166 (7.38)
Hypertension	2598 (23.2)	1153 (25.0)	1265 (30.3)	159 (7.07)
Obesity	2333 (20.8)	838 (18.2)	1345 (32.2)	137 (6.09)
Solid organ transplant	36 (0.32)	14 (0.30)	21 (0.50)	0
NDI, median (IQR)	0.30 (−0.67 to 1.30)	−0.11 (−0.70 to 0.56)	0.82 (0.08 to 1.76)	0.34 (−0.25 to 1.33)

Abbreviations: COPD, chronic obstructive pulmonary disease; ECI, Agency for Healthcare Research and Quality Elixhauser Comorbidity Index; IQR, interquartile range; NDI, neighborhood deprivation index.

^a^ Overall, 81 individuals were missing race data and 3 individuals were missing age data.

^b^ ECI was calculated based on *International Statistical Classification of Diseases and Related Health Problems, Tenth Revision* diagnosis codes; therefore, no ECI data were available on those currently hospitalized.

Most patients (7139 [63.7%]) required hospitalization (Table 2). Overall, 2812 Black patients (39.4%) were admitted to a hospital. Compared with hospitalized White patients, hospitalized Black patients were more likely to present with a temperature of at least 38 °C (432 of 1952 [22.1%] vs 535 of 1668 [32.1%]) and respiratory rate of at least 24 breaths per minute (579 of 1956 [29.6%] vs 570 of 1674 [34.1%]) and were less likely to have an oxygen saturation less than 94% (801 of 1959 [40.9%] vs 585 of 1674 [34.9%]). Hospitalized Black patients had more comorbid conditions than hospitalized White patients (median [IQR] ECI score, 34 [19-55] vs 32 [15-54]) and a higher prevalence (ie, >10% difference) of chronic kidney disease (808 [28.7%] vs 572 [18.3%]), diabetes (1156 [41.1%] vs 920 [29.4%]), and obesity (1134 [40.3%] vs 767 [24.5%]).

**Table 2.** Characteristics of Adults With Coronavirus Disease 2019 Admitted to 92 Hospitals in a Multistate US Health Care System

Characteristic	Patients, No. (%)			
	All (N = 7139)	White (n = 3128)	Black (n = 2812)	With other race (n = 1118)^a^
Age, median (IQR), y^b^	68 (56-79)	72 (60-83)	66 (54.25-76)	61 (49-74)
Sex				
Women	3470 (48.6)	1503 (48.0)	1469 (52.2)	457 (40.9)
Men	3664 (51.3)	1624 (51.9)	1342 (47.7)	658 (58.9)
Insurance				
Commercial	1457 (20.4)	609 (19.5)	595 (21.2)	231 (20.7)
Medicare	4211 (59.0)	2099 (67.1)	1651 (58.7)	424 (37.9)
Medicaid	1256 (17.6)	337 (10.8)	514 (18.3)	390 (34.9)
Other	165 (2.31)	59 (1.89)	37 (1.32)	64 (5.72)
Unknown	50 (0.70)	24 (0.77)	15 (0.53)	9 (0.81)
Comorbidities				
ECI, median (IQR)^c^	32 (15-54)	32 (15-54)	34 (19-55)	30 (3-47)
Asthma	441 (6.18)	147 (4.70)	264 (9.39)	27 (2.42)
Cancer	295 (4.13)	138 (4.41)	141 (5.01)	15 (1.34)
Chronic kidney disease	1465 (20.5)	572 (18.3)	808 (28.7)	74 (6.62)
Chronic liver disease	304 (4.26)	146 (4.67)	131 (4.66)	23 (2.06)
COPD	879 (12.3)	466 (14.9)	379 (13.5)	31 (2.77)
Congestive heart failure	1020 (14.3)	467 (14.9)	497 (17.7)	46 (4.11)
Coronary artery disease	1087 (15.2)	560 (17.9)	470 (16.7)	50 (4.47)
Diabetes	2237 (31.3)	920 (29.4)	1156 (41.1)	142 (12.7)
Hypertension	2120 (29.7)	955 (30.5)	1023 (36.4)	126 (11.3)
Obesity	2044 (28.6)	767 (24.5)	1134 (40.3)	130 (11.6)
Solid organ transplant	32 (0.45)	13 (0.42)	18 (0.64)	0
NDI, median (IQR)	0.23 (-0.42-1.33)	-0.21 (-0.73-0.55)	0.75 (0.01-1.86)	0.23 (-0.36-1.33)
First documented vital sign, No./total No. (%)^d^				
Respiratory rate ≥24 breaths/min	1225/3936 (31.1)	579/1956 (29.6)	570/1674 (34.1)	64/257 (24.9)
Temperature ≥38 °C	1064/3928 (27.1)	432/1952 (22.1)	535/1668 (32.1)	80/257 (31.1)
Oxygen saturation <94%	1504/3941 (38.2)	801/1959 (40.9)	585/1674 (34.9)	100/257 (38.9)
Level of hospital care				
Non-ICU care	4273 (59.9)	1816 (58.1)	1710 (60.8)	703 (62.9)
ICU care	2866 (40.1)	1312 (41.9)	1102 (39.2)	415 (37.1)
ICU care and ventilator	2268 (31.8)	1064 (34.0)	877 (31.2)	301 (26.9)
ICU care and no ventilator	598 (8.4)	248 (7.9)	225 (8.0)	114 (10.2)
Length of stay, median (IQR), d	7 (4-12)	7 (4-12)	6 (4-12)	7 (4-12)

Abbreviations: COPD, chronic obstructive pulmonary disease; ECI, Agency for Healthcare Research and Quality Elixhauser Comorbidity Index; ICU, intensive care unit; IQR, interquartile range; NDI, neighborhood deprivation index.

^a^ Overall, 81 individuals were missing race data.

^b^ A total of 3 individuals were missing age data.

^c^ ECI was calculated based on *International Statistical Classification of Diseases and Related Health Problems, Tenth Revision* diagnosis codes; therefore, no ECI data were available on those currently hospitalized.

^d^ First documented vital sign was collected from the electronic health record when available, representing approximately 55% of all hospitalized patients.

**Figure 1.**
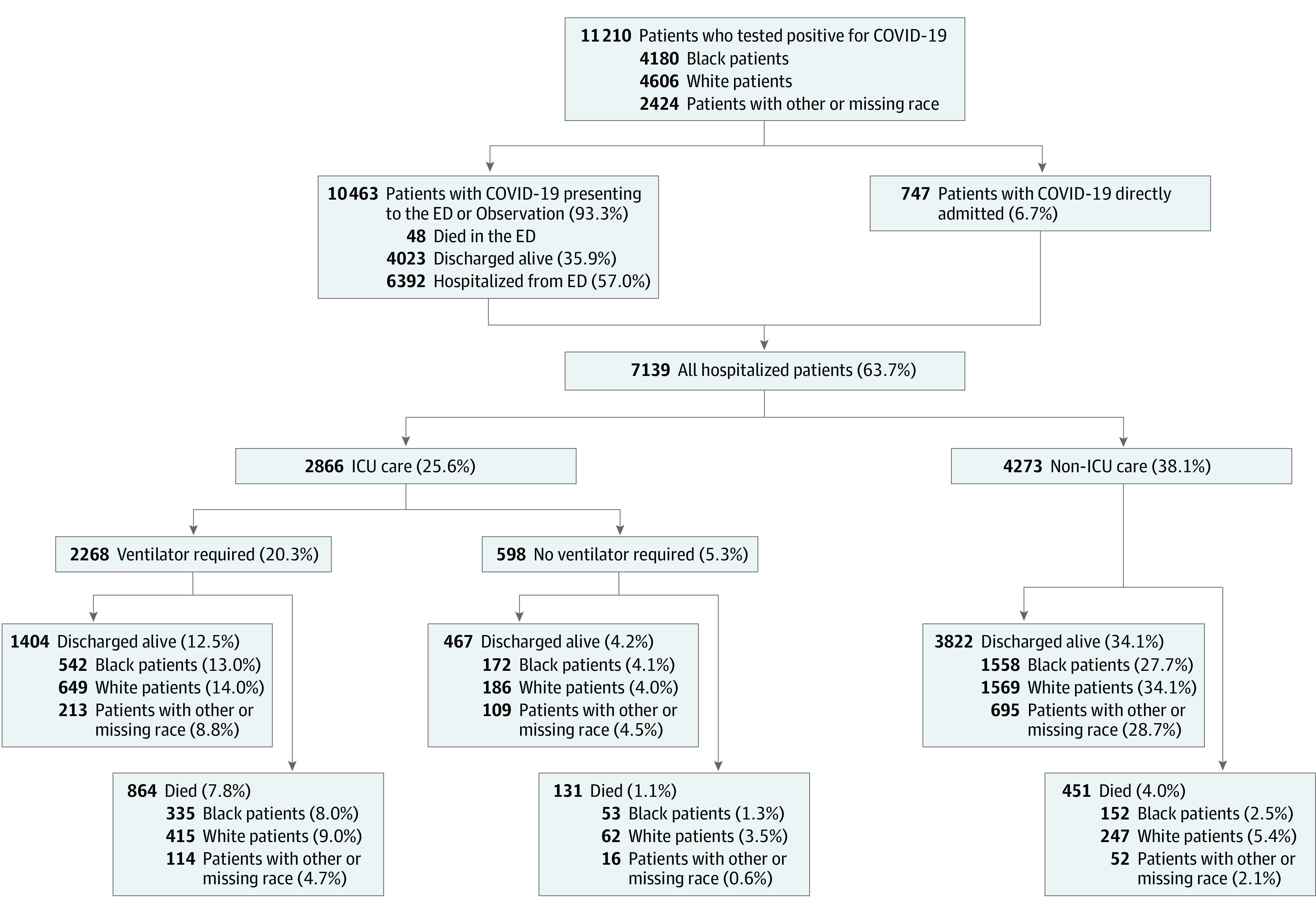
Health Care Utilization and All-Cause, In-Hospital Mortality for Patients With Confirmed Coronavirus Disease 2019 (COVID-19) ED indicates emergency department; ICU, intensive care unit.

**Figure 2.**
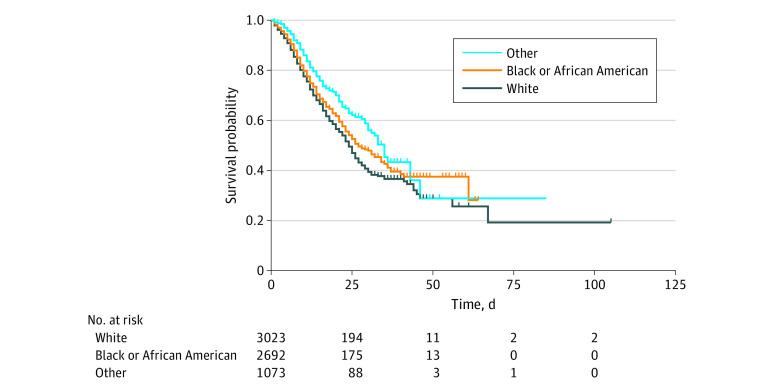
Kaplan-Meier Survival Curve for Race Among Adults Hospitalized With Coronavirus Disease 2019 in a Multistate US Health Care System

Among hospitalized patients, 2866 (40.1%) were admitted to the ICU, and 2268 (31.8%) received mechanical ventilation. Black and White patients used the ICU and invasive mechanical ventilation at similar rates (1102 [39.2%] vs 1312 [41.9%] and 877 [31.2%] vs 1064 [34.0%], respectively). Overall, all-cause, in-hospital mortality was 20.3% (1446 patients), 34.7% among those with an ICU stay (995 of 2866 patients), and 38.1% for those receiving mechanical ventilation (864 of 2268 patients) (Figure 1). Mortality among White and Black patients was 23.1% (724 of 3128) and 19.2% (540 of 2812), respectively. Among those with an ICU stay, 36.4% of White patients (477 of 1312) and 35.2% of Black patients (388 of 1102) died. For those receiving mechanical ventilation, 39.0% of White patients (415 of 1064) and 38.2% of Black patients (335 of 877) died. Of the 7139 hospitalized patients, 53 (0.7%) were still in the hospital as of June 25, 2020, and not included in time-to-event analyses. Table 3 shows the Cox proportional hazards mixed effects models for patient characteristics associated with all-cause, in-hospital mortality. In the final adjusted model (model 3), persons older than 85 years, aged 65 to 84 years, and aged 50 to 64 years had 3.96 (95% CI, 2.82-5.55), 2.38 (95% CI, 1.73-3.26), and 1.36 (95% CI, 1.11-1.67), respectively, times the risk of death compared with those aged 18 to 49 years. Male patients had 1.23 (95% CI 1.11-1.36) times the risk of death compared with female patients. Patients with Medicare insurance (HR, 1.47; 95% CI, 1.08-2.00) and individuals whose insurance coverage was unknown (HR, 2.17; 95% CI, 1.32-3.57) had higher risk of mortality than those with commercial insurance. Patients with chronic kidney disease (HR, 1.15; 95% CI, 1.03-1.30) and coronary artery disease (HR, 1.18; 95% CI, 1.07-1.32) had higher mortality than those without these conditions. Adjusting for patient sociodemographic and clinical factors, race was not significantly associated with an increased risk of death (HR, 0.93; 95% CI, 0.83-1.09) (Figure 2). Sensitivity analysis modeling categorical age groups vs continuous age revealed a nonlinear association between age and mortality, with older age groups having a higher risk of death than a corresponding risk calculated from the continuous age model estimate. Additionally, both approaches yielded nearly identical estimates for all covariates.

**Table 3.** Hazard Ratios for All-Cause, In-Hospital Death Among Patients With Coronavirus Disease 2019 Admitted to 92 Hospitals in a Multistate US Health Care System

Characteristic	Hazard ratio (95% CI)^a^		
	Model 1	Model 2	Model 3
Race			
White	1 [Reference]	1 [Reference]	1 [Reference]
Black	0.86 (0.76-0.96)	0.93 (0.80-1.09)	0.93 (0.80-1.09)
Other	0.62 (0.52-0.73)	0.77 (0.55-1.07)	0.83 (0.64-1.08)
Age, y			
18-49	1 [Reference]	1 [Reference]	1 [Reference]
50-64	1.54 (1.17-2.03)	1.40 (1.13-1.73)	1.36 (1.11-1.67)
65-84	3.36 (2.61-4.34)	2.44 (1.79-3.33)	2.38 (1.73-3.26)
≥85	5.65 (4.32-7.38)	4.00 (2.95-5.47)	3.96 (2.82-5.55)
Male sex	1.09 (0.98-1.21)	1.24 (1.11-1.38)	1.23 (1.11-1.36)
Insurance			
Commercial	1 [Reference]	1 [Reference]	1 [Reference]
Medicare	2.70 (2.26-3.22)	1.57 (1.17-2.10)	1.47 (1.08-2.00)
Medicaid	1.07 (0.85-1.36)	1.17 (0.93-1.46)	1.17 (0.92-1.47)
Other	1.21 (0.72-2.03)	1.38 (0.71-2.66)	1.40 (0.78-2.53)
Unknown	2.10 (1.07-4.13)	2.11 (1.27-3.51)	2.17 (1.32-3.57)
NDI		1.02 (0.90-1.16)	1.01 (0.89-1.15)
Comorbidities			
ECI score	1.01 (1.01-1.01)	1.00 (1.00-1.01)	1.00 (1.00-1.00)
Asthma	0.73 (0.57-0.94)	NA	0.91 (0.74-1.12)
Cancer	1.28 (1.03-1.60)	NA	1.15 (0.91-1.47)
Chronic kidney disease	1.48 (1.33-1.66)	NA	1.15 (1.03-1.30)
Chronic liver disease	1.17 (0.96-1.42)	NA	NA
COPD	1.55 (1.36-1.77)	NA	1.22 (0.94-1.57)
Congestive heart failure	1.55 (1.37-1.76)	NA	1.05 (0.91-1.22)
Coronary artery disease	1.71 (1.51-1.92)	NA	1.18 (1.07-1.32)
Diabetes	1.17 (1.05-1.30)	NA	1.08 (0.85-1.36)
Hypertension	1.06 (0.94-1.18)	NA	NA
Obesity	0.86 (0.77-0.97)	NA	0.97 (0.81-1.16)
Solid organ transplant	1.59 (0.90-2.81)	NA	NA

Abbreviations: COPD, chronic obstructive pulmonary disease; ECI, Agency for Healthcare Research and Quality Elixhauser Comorbidity Index; NA, not applicable; NDI, neighborhood deprivation index.

^a^ All models were Cox proportional hazards models with mixed effects using hospital as random effects and patient characteristics as fixed effects. Model 1 presents unadjusted hazard ratios for all patient characteristics. Model 2 presents adjusted hazard ratios for a priori patient characteristics of interest (race, age, sex, insurance, and ECI and NDI scores). Model 3 presents adjusted hazard ratios for covariates in model 2 as well asthma, cancer, chronic kidney disease, COPD, congestive heart failure, coronary artery disease, diabetes, and obesity, which were statistically significant in univariable analyses.

## Discussion

In our cohort of 92 hospitals across 12 states, there was no statistically significant difference in all-cause, in-hospital mortality between White and Black patients after adjusting for other factors. Overall all-cause, in-hospital mortality was 20.3%; it was 38.1% among patients receiving mechanical ventilation, similar to findings from other US studies.^3,7,10,11,16^ Consistent with prior research, older age and male sex were significantly associated with risk of death.^4,6,7,10,11,17^

Current data indicate that Black residents represent a disproportionate share of US COVID-19 cases and deaths.^3,4,5^ Living conditions (eg, residence in densely populated areas; multigeneration households; the presence of racial segregation; overrepresentation in jails, prisons, and detention centers), work circumstances (eg, higher rates of essential workers; lack of paid sick leave), and underlying health conditions (eg, lack of health insurance; higher rates of certain comorbidities; chronic and toxic stress associated with stigma and systemic inequalities) may help explain the disproportionate burden of illness and death among patients who belong to minority groups.^9,18^ Our findings confirm data from Louisiana (a cohort of patients seeking care at a New Orleans–based integrated delivery health system)^10^ and Georgia (a convenience sample of hospitalized adult patients in metropolitan Atlanta and southern Georgia),^11^ which reported no difference in mortality by race among hospitalized patients with COVID-19. Taken together, these findings suggest that while Black US residents may be at higher risk of contracting COVID-19 and represent a disproportionate share of COVID-19 death, mortality for those able to access hospital care does not differ from White patients.

In our sample, older age was the strongest independent risk factor for death, with White patients a median of 6 years older than Black patients. This age difference between patients admitted to the hospital with COVID-19 is consistent with other studies.^3,10,11^ Similar to prior reports, we noted higher rates of diabetes, hypertension, chronic kidney disease, and obesity among Black patients.^10,11^ Additionally, Black patients used the ICU and invasive mechanical ventilation at similar rates as White patients (1102 [39.2%] vs 1312 [41.9%] and 877 [31.2%] vs 1064 [34.0%], respectively). This is consistent with reports from Georgia but different from Louisiana data, which showed higher ICU and invasive mechanical ventilation among Black patients.^10,11^

### Limitations

This study has several limitations. First, our study focused on individuals able to access hospital care. We did not observe patients before or after discharge. Mortality may vary when accounting for death prior to and after hospitalization. Second, collection of ethnicity data varied by hospital. Therefore, we were unable to examine the association of ethnicity with mortality. Third, neighborhood deprivation was measured at the zip code level and may not reflect individual factors. Similarly, ECI is based on *ICD*-*10* diagnosis codes, which may not fully reflect patients’ comorbidities. It is possible that documentation of diagnosis codes for some patients was incomplete. Fourth, while our hospitals are located in diverse settings across multiple states, our experience may not be representative of other hospitals.

## Conclusions

In this analysis of patients with COVID-19, higher risk of all-cause in-hospital mortality was associated with older age, male sex, Medicare insurance, coexisting chronic kidney disease, and coronary artery disease. No statistically significant difference by race was observed. Additional studies examining COVID-19 mortality by race, accounting for death prior to and after hospitalization, are needed.
